# The effects of stimulus distribution form during trace conditioning

**DOI:** 10.1080/17470218.2017.1367017

**Published:** 2018-01-01

**Authors:** Charlotte Bonardi, Dómhnall J Jennings

**Affiliations:** 1 School of Psychology, University of Nottingham, Nottingham, UK; 2 Institute of Neuroscience, Newcastle University, Newcastle upon Tyne, UK

**Keywords:** Trace conditioning, distribution form, rats, timing, associative strength

## Abstract

Three experiments examined the effect of distribution form of the trace interval on trace conditioning. In Experiments 1 and 2, two groups of rats were conditioned to a fixed-duration conditioned stimulus (CS) in a trace interval procedure; rats in Group Fix received a fixed-duration trace interval, whereas for rats in Group Var the trace interval was of variable duration. Responding during the CS was higher in Group Var than in Group Fix, whereas during the trace interval this difference in responding reversed—Group Fix showed higher response rates than Group Var. Experiment 3 examined whether the greater response rate observed during the CS in Group Var was due to a performance effect or the acquisition of greater associative strength by the CS. Following trace conditioning, the rats from Experiment 1 underwent a second phase of delay conditioning with the same CS; a 5-s auditory stimulus was presented in compound with the last 5 s of the 15-s CS, and the unconditioned stimulus (US) was delivered at the offset of the CSs. On test with the auditory stimulus alone, subjects in Group Var showed lower response rates during the auditory stimulus than subjects in Group Fix. We interpreted these findings as evidence that the superior responding in Group Var during the CS was a result of it acquiring greater associative strength than in Group Fix.

## Introduction

Classical conditioning tasks, in which a neutral conditioned stimulus (CS) signals delivery of an unconditioned stimulus (US) of motivational value, are routinely used to examine the mechanisms underlying learning in many species. In the most common version of the task, the CS is of the same temporal duration on every trial and signals a punctate US—so CS onset can be used by the animal to accurately predict the *time* of US occurrence. This suggestion is supported by a large body of evidence showing timing in conditioning tasks; for example, in rodents, the rate of conditioned responding typically increases steadily over the course of the CS to reach a maximum at the point of US delivery (Roberts, 1981).

Although conditioning and timing are observed in the same task, traditionally they have not been explained within the same theoretical framework (Kirkpatrick, 2014). However, more recent hybrid theories have tried to explain conditioning using theories of timing (e.g., Balsam & Gallistel, 2009; Gallistel & Gibbon, 2000; Gibbon & Balsam, 1981). One salient feature of many such accounts, which sets them apart from more orthodox conditioning theories, is their rejection of the importance of the *trial*. For standard conditioning theories, the trial—a pairing of CS and US—is a unit of learning that increments *associative strength*, reflecting the degree to which presentation of the CS can evoke activation of the US and elicit the conditioned response (CR). They assume that more complex phenomena, such as the sensitivity to CS/US correlation, are emergent properties of trial-by-trial learning (e.g., Mackintosh, 1975; Pearce & Hall, 1980; Rescorla & Wagner, 1972; Wagner, 1981). In contrast, hybrid timing theories often adopt an information processing approach, positing that animals are sensitive to statistical properties of the environment, such as reinforcement rate and stimulus informativeness. Once they reach a certain threshold—for example, the reinforcement rate during the CS is substantially higher than that in the background, indicating that the CS signals an increase in likelihood of the US—a *decision* is made to respond (cf., Gibbon & Balsam, 1981). Critically, these properties are derived from a broader set of events than a single trial.

But although this difference in the importance attributed to the trial should help discriminate between the two approaches, in practice it has proved difficult to do so because the two classes of theory use different independent variables. Conditioning theories stress that the level of CR is the key indicator of associative strength, while speed of acquisition, indexing the point of decision to respond, is the main measure adopted by hybrid theories. For example, we conducted a study that examined whether conditioning differed between fixed-duration CSs and CSs that varied in duration trial-by-trial but overall were matched in *mean* duration to the fixed CS (Jennings, Alonso, Mondragòn, Franssen, & Bonardi, 2013). We found that speed of acquisition of the CR was generally higher in the variable-duration stimulus, contrary to the predictions of hybrid theories; such a result falls beyond the scope of conditioning theories.

Moreover, differences in conditioned responding were also observed: The level of CR was higher in the fixed-duration cue than the variable CS, and this was not simply a performance effect. For example, it might for some reason have been *easier* to respond during a fixed-duration CS; however, we observed higher levels of responding to a cue trained with a fixed duration even when the animals were tested under identical conditions (Jennings et al., 2013). Furthermore, we also demonstrated that fixed-duration cues produce better overshadowing and better blocking than their variable counterparts (Bonardi, Mondragón, Brilot & Jennings, 2015; Jennings & Bonardi, 2017). Conditioning theories would interpret these findings as evidence that fixed-duration cues acquire *more associative strength* than variable CSs.

Although it is possible to derive an explanation of this effect from conditioning theories (e.g., Jennings et al., 2013), such accounts are arguably a little post hoc and are designed to address a relatively narrow range of findings. It would therefore be helpful to further define the empirical boundary conditions under which these differences are observed. For example, CSs that are fixed and variable differ not only in their temporal nature but also in the degree to which their *onset* predicts the time of reinforcement: The onset of a fixed CS is an accurate predictor of reinforcement, but the onset of a variable cue is not. Either could be responsible for the apparent difference in associative strength seen in the two types of stimulus. The present experiments attempted to dissociate these possibilities.

Two groups of rats were trained on an appetitive trace conditioning task in which the CS was of a fixed duration. The groups differed in whether the *trace* interval was fixed or variable. When the trace interval was fixed, CS onset gave accurate information about the time of US delivery, whereas when it was variable it would not. This allowed us to differentiate the temporal nature of the CS and the informativeness of CS onset as potential explanations of our previous findings.

## Experiment 1

Two groups of rats, Group Fix and Group Var, were trained on a trace conditioning task with a visual CS; for Group Fix, the duration of the trace interval was fixed, whereas for Group Var, it was variable but with the same mean duration as in Group Fix.

### Subjects

The subjects were 16 Lister hooded rats (Harlan, UK) with a mean free-feeding weight of 287 g (range: 270-305 g), housed in pairs in plastic tub cages with sawdust bedding. They were deprived to 85% of their ad lib weight before the start of the experiment and maintained at this level (with regular adjustments for natural growth rate) by being fed a restricted amount of food at the end of each session. Water was freely available in the home cages. They were maintained on a 12-hr light/dark cycle, the light period starting at 7 a.m.; the temperature was maintained at 21°C (±1°C) and the humidity at 60% (±10%).

### Apparatus

The apparatus comprised eight identical chambers (20 cm × 24 cm × 30 cm), each in a ventilated, noise-attenuating box (74 cm × 38 cm × 60 cm; MED Associates). Each chamber was equipped with a houselight and a food cup; two 2.8 W jewel lights, one 2.5 cm to each side of the food cup; and a speaker, on the right side of the wall opposite to the food cup. A pellet dispenser (Model ENV-203) delivered 45 mg TestDiet pellets (MLab Rodent Tablets) into the food cup. Each head entry into the food cup was detected by a light-emitting diode (LED) photocell and recorded as a single response. Med-PC for Windows (Tatham & Zurn, 1989) controlled experimental events; trials of the same duration were delivered at the same time across experimental chambers. The time of occurrence of each stimulus onset, stimulus termination, food delivery, and head entry response was recorded with a resolution of 10 ms.

### Procedure

*Phase 1* comprised eight sessions of 24 trials (aside from the first session which had 30 trials). Each trial consisted of a 30-s pre-CS period followed by the CS, a 30-s illumination of the two jewel lights. In Group Fix, CS offset was followed by delivery of a single food pellet after a trace interval of 30 s; trials in Group Var were identical except that the trace interval was of a variable duration drawn from an exponential distribution with a mean of 30 s. The intertrial interval (ITI), which ran from food delivery to the start of the next pre-CS period, comprised a fixed interval of 60 s plus a variable interval, again from an exponential distribution, with a mean of 90 s.

As will be seen below, numerical differences began to emerge between the two groups, but these were quite modest, which we attributed to the relatively long, 30-s, duration of the CS and trace interval; thus, in Phases 2 and 3, we reduced the duration of these intervals in an attempt to magnify the size of the observed effects.

*Phase 2* was identical to *Phase 1* except that the trace interval was reduced to 15 s in Group Fix and a mean of 15 s in Group Var. There were 12 sessions in this stage.

*Phase 3* was identical to *Phase 2* except that the CS duration was reduced to 15 s in both groups. There were 12 sessions in this stage.

### Data treatment

Mean rates of responding during pre-CS, CS, and trace intervals in each session were computed for each rat, and then CS and trace *difference* scores—reflecting the degree to which responding during CS and trace was higher than during the background—were calculated by subtracting the pre-CS rate from the CS and the trace interval response rates, respectively. The data were analysed in four-session blocks.

In addition, the response rates in each 1-s bin of the CS and trace intervals, averaged over each phase, were computed. The rates were then normalised (divided by the total number of responses for that rat for that session) to give the percentage of responses in each 1-s time bin, and a linear function fitted to each normalised response function. The slope of this function allowed us to characterise the distribution of responding over the course of the CS (Jennings, Bonardi, & Kirkpatrick, 2007; cf., Kirkpatrick & Church, 2000). Positive slopes result from more head entry responses at the end of the interval than at the beginning, whereas negative slopes indicate the opposite (Jennings & Kirkpatrick, 2006; Kirkpatrick & Church, 2000). As timing would produce an increase in responding as the US approaches, positive slopes can be taken to indicate timing. In addition, to examine the distribution of conditioned responding over the course of the CS and trace intervals, response rates in each 1 s bins were averaged into 3 s bins. This yields 10 bins each for the 30-s CS and 30-s trace in *Phase 1*, 10 bins for the 30-s CS and five bins for the 15-s trace in *Phase 2*, and five bins each for the 15-s CS and 15-s trace in *Phase 3*.

Results were analysed using mixed analysis of variance (ANOVA); significant two-way interactions were examined with simple main effects analysis using the pooled error term and significant three-way interactions with further two-way ANOVAs. The significance level was set at *p* < .05, and $\eta_{p}^{2}$ was reported for significant effects and interactions.

### Results

#### Difference scores

**Figure 1.**
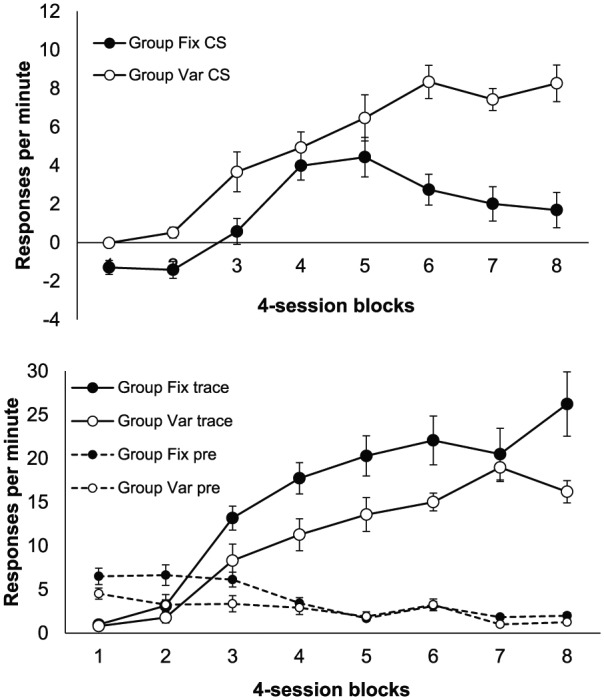
Group mean difference scores for the CS (upper panel) and the trace interval (lower panel) in the three training phases of Experiment 1; the lower panel also shows group mean rates of pre-CS responding. Data are reported in four-session blocks; error bars show standard error of the mean.

These are shown in Figure 1; scores for the CS are shown in the upper panel and those for the trace interval, and also rates of pre-CS responding, in the lower panel. It is evident that although responding was higher in Group Fix during the trace interval, at least from the start of *Phase 2* (Block 3, lower panel), the upper panel suggests the opposite was true during the CS, especially in *Phase 3* (Blocks 6, 7 and 8).

An ANOVA was conducted on these difference scores, with group (Fix or Var), interval (CS or trace), and block as factors; this revealed a significant three-way interaction between these factors, *F*(7, 98) = 8.81, *p* < .001, mean squared error (*MSE*) = 5.29, $\eta_{p}^{2}=.39$, and so ANOVAs were then conducted on the difference scores for CS and trace interval data separately, with group and block as factors.

ANOVA on the CS scores revealed main effects of both group and block, *F*(1, 14) = 19.07, *p* = .001, *MSE* = 18.90, $\eta_{p}^{2}=.58$ and *F*(7, 98) = 36.09, *p* < .001, *MSE* = 2.97, $\eta_{p}^{2}=.72$, respectively, and an interaction, *F*(7, 98) = 6.47, *p* < .001, *MSE* = 2.97, $\eta_{p}^{2}=.32$. Difference scores were higher in Group Var on Blocks 3, 6, 7, and 8 (smallest, *F*(1, 112) = 7.72, *p* = .006, *MSE* = 4.96, for Block 3). ANOVA on the trace scores also revealed main effects of both group and block, *F*(1, 14) = 5.24, *p* = .038, *MSE* = 139.44, $\eta_{p}^{2}=.27$ and *F*(7, 98) = 74.47, *p* < .001, *MSE* = 13.01, $\eta_{p}^{2}=.84$, respectively, and an interaction, *F*(7, 98) = 3.65, *p* = .002, *MSE* = 13.01, $\eta_{p}^{2}=.21$; scores were higher in Group Fix on Blocks 4, 5, 6, and 8 (smallest, *F*(1, 112) = 5.80, *p* = .018, *MSE* = 28.81, for Block 4).

The pre-CS rates, also shown in the lower panel, were slightly higher in Group Fix in Blocks 1 to 3, but this difference quickly dissipated. A two-way ANOVA with group and block as factors revealed a main effect of block, *F*(7, 98) = 19.19, *p* < .001, *MSE* = 2.18, $\eta_{p}^{2}=.58$, and a Group × Block interaction, *F*(7, 98) = 3.24, *p* = .004, *MSE* = 2.18, $\eta_{p}^{2}=.19$; the groups differed on Blocks 1, 2, and 3 only (smallest, *F*(1, 112) = 3.98, *p* = .048, *MSE* = 3.96, for Block 1). The higher rate of pre-CS responding in Block 3 would have differentially reduced the CS difference score in Group Fix and so could have been responsible for this group’s lower CS difference scores on this block. But the pre-CS rates did not differ from Block 4 onwards (*F*s < 1), and so group differences from this point cannot be attributed to differences in background responding.

In summary, the groups showed opposite patterns of responding in the CS and trace intervals, with Group Fix responding more during the trace interval but *less* during the CS. The higher responding during the CS in Group Var was only reliable in *Phase 3*, whereas during the trace interval, responding was higher in Group Fix during most of *Phases 2* and *3*.

### Timing

#### Response functions

**Figure 2.**
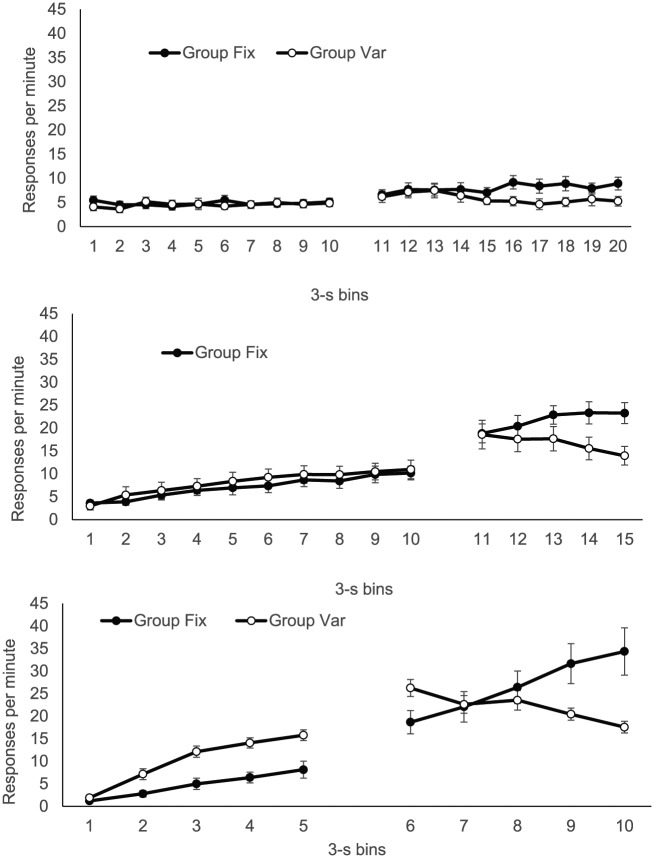
Group mean response rates in each 3-s bin of the CS and trace interval during each of the three phases of Experiment 1; upper, middle, and lower panels show data from Phases 1, 2, and 3, respectively. The CS is 10 bins in Phases 1 and 2 and five bins in Phase 3; the trace is 10 bins in Phase 1 and five bins in Phases 2 and 3. Error bars show standard error of the mean.

The group mean distribution of responding in 3 s bins, over both CS and trace intervals, is presented separately for each phase in Figure 2. In *Phase 1*, response rates remained low and steady over the CS (upper panel, Bins 1-10), giving little sign that the rats knew food was closer to being delivered at CS offset; there was also little difference between the groups. In *Phase 2* (centre panel, Bins 1-10), there was a modest increase in responding over the CS in both groups, with Group Var showing very slightly higher rates, but by *Phase 3* (lower panel, Bins 1-5), when the CS duration was reduced to 15 s, there was substantially more responding at the end of the CS, providing clearer evidence of timing; by this point, rates were clearly higher in Group Var.

During the trace interval, response rates were generally higher in Group Fix in all phases (upper panel, Bins 11-20; centre panel, Bins 11-15; lower panel, Bins 6-10 for Phases 1, 2, and 3, respectively). Notably, in *Phase 3*, the higher responding evident in Group Var during the CS persisted for the first bin of the trace interval (Bin 6), before reversing from Bin 8. In addition, during the trace interval, there was evidence of more timed responding as the US approached in *Phase 2* (centre panel, Bins 11-15) and *Phase 3* (lower panel, Bins 6-10)—but only in Group Fix; in Group Var, response rates, if anything, *decreased* over the trace interval.

These impressions were broadly supported by the analyses. In *Phase 1*, ANOVA with group, interval (CS/trace), and bin as factors revealed a significant three-way interaction, *F*(9, 126) = 3.97, *p* < .001, *MSE* = 1.43, $\eta_{p}^{2}=.22$. To explore this interaction, separate ANOVAs were conducted on the CS and trace data. The CS analysis revealed a significant Group × Bin interaction, *F*(9, 126) = 2.25, *p* = .023, *MSE* = 0.84, $\eta_{p}^{2}=.14$, but the groups did not differ on any bin (largest, *F*(1, 140) = 1.38, *p* = .25, *MSE* = 5.45, for Bin 1). Parallel analysis of the trace data also yielded a significant interaction, *F*(9, 126) = 4.97, *p* < .001, *MSE* = 1.92, $\eta_{p}^{2}=.26$, and here, there was higher responding in Group Fix in Bins 16, 17, 18, and 20 (smallest, *F*(1, 140) = 4.40, *p* = .038, *MSE* = 12.13, for Bin 20).

Because of the difference in CS and trace duration (30 and 15 s, respectively) during *Phase 2*, here the analysis was conducted with three levels of interval (first half of CS, second half of CS, trace) each corresponding to five 30 s bins. Again, the three-way interaction was significant, *F*(8, 112) = 7.60, *p* < .001, *MSE* = 2.65, $\eta_{p}^{2}=.35$. Separate ANOVAs for the first and second half of the CS revealed only main effects of bin, *F*(4, 56) = 29.11, *p* < .001, *MSE* = 1.66, $\eta_{p}^{2}=.68$ and *F*(4, 56) = 11.74, *p* < .001, *MSE* = 1.10, $\eta_{p}^{2}=.46$, respectively. In contrast, the trace interval ANOVA revealed a significant Group × Bin interaction, *F*(4, 56) = 12.57, *p* < .001, *MSE* = 4.26, $\eta_{p}^{2}=.47$; there was greater responding in Group Fix in Bins 14 and 15 (smallest, *F*(1, 70) = 5.07, *p* < .028, *MSE* = 47.84, for Block 14).

*Phase 3* analysis, with group, interval (CS/trace), and bins as factors, revealed a significant three-way interaction, *F*(4, 56) = 27.00, *p* < .001, *MSE* = 11.32, $\eta_{p}^{2}=.66$. ANOVA of the CS data yielded a Group × Bin interaction, *F*(4, 56) = 13.32, *p* < .001, *MSE* = 2.75, $\eta_{p}^{2}=.49$, and higher responding in Group Var on Bins 3, 4, and 5 (smallest, *F*(1, 70) = 19.61, *p* < .001, *MSE* = 10.38, for Block 3). ANOVA of the trace data also revealed a significant interaction, *F*(4, 56) = 20.92, *p* < .001, *MSE* = 17.70, $\eta_{p}^{2}=.60$, and higher responding in Group Fix on Bins 9 and 10 (smallest, *F*(1, 70) = 6.69, *p* = .01, *MSE* = 75.19, for Block 9).

#### Slopes

**Figure 3.**
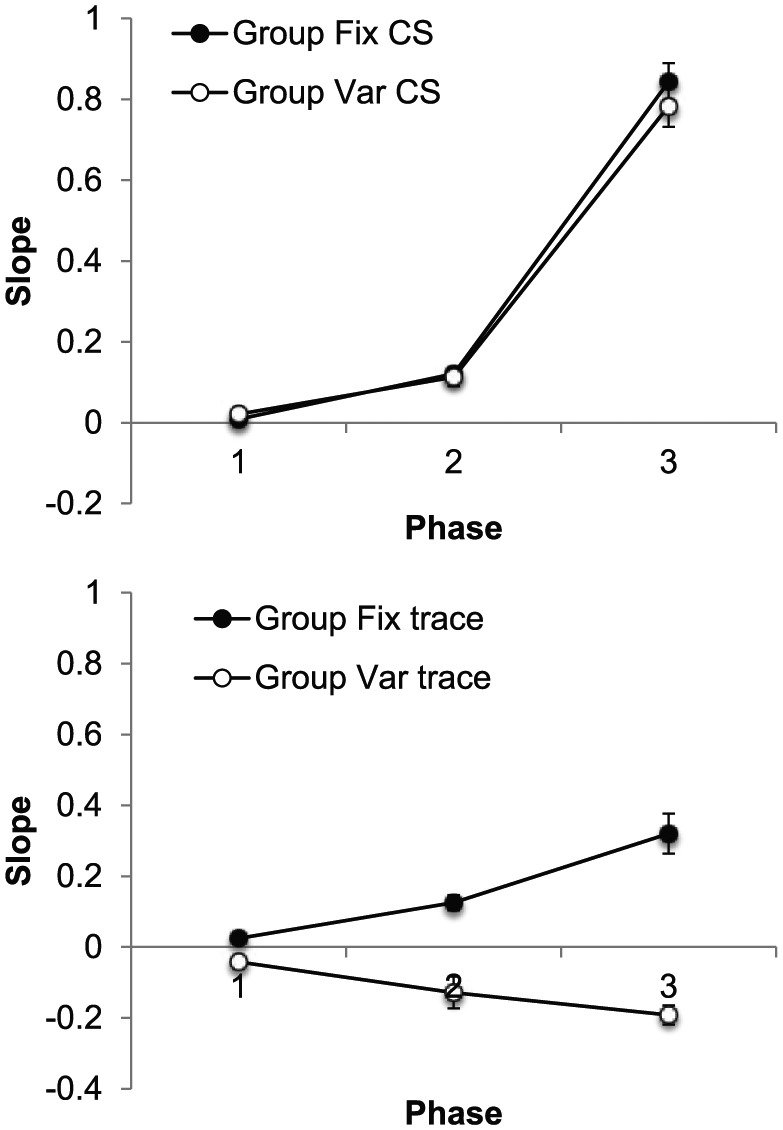
Group mean values of slope for the CS (upper panel) and trace interval (lower panel) in each of the three training phases of Experiment 1. Error bars show standard error of the mean.

The slope data are presented in Figure 3, separately for each phase; CS slopes are shown in the upper panel and trace slopes in the lower. The upper panel shows CS slopes increased markedly across phases, but there was little sign of a difference between the groups; in contrast, during the trace intervals, slopes in Group Fix increased across phases and were markedly higher than those in Group Var, which decreased. ANOVA with group, interval (CS/trace), and phase as factors revealed a significant three-way interaction, *F*(2, 28) = 7.67, *p* = .002, *MSE* = 0.009, $\eta_{p}^{2}=.36$, and so separate ANOVAs were conducted on CS and trace slopes.

ANOVA on the CS data, with group and phase as factors, revealed an effect of phase, *F*(2, 28) = 485.21, *p* < .001, *MSE* = 0.006, $\eta_{p}^{2}=.97$; the effect and interaction involving group were not significant, *F*s < 1. In contrast, ANOVA of the trace slopes revealed a significant Group × Phase interaction, *F*(2, 28) = 24.12, *p* < .001, *MSE* = 0.008, $\eta_{p}^{2}=.63$, and the groups differed on *Phases 2* and *3* (smallest, *F*(1, 42) = 28.15, *p* < .001, *MSE* = 0.009, for *Phase 2*). In addition, slopes increased across phases in Group Fix, *F*(2, 28) = 21.76, *p* < .001, *MSE* = 0.008, but decreased in Group Var, *F*(2, 28) = 5.47, *p* < .01, *MSE* = 0.008.

### Discussion

Previous work in our laboratory has shown that rats show higher levels of conditioned responding to a CS when it is of a fixed duration than when its duration is variable (Jennings et al., 2013). In the present experiment, the CSs in both groups were fixed, but the trace intervals were not, being fixed in Group Fix and variable in Group Var; thus, the CSs signalled fixed and variable intervals to reinforcement, respectively. But although this *might* be expected to produce the same effect on responding as a fixed and variable CS, it did not. In fact, the opposite effect on responding was seen during the CS—rates were higher in Group Var, and it was only in the trace interval that the advantage in responding during the fixed interval we have seen in previous experiments was evident. The aim of Experiment 2 was to try and replicate this unexpected result under conditions in which both CS and trace interval duration were constant throughout training.

## Experiment 2

### Subjects and apparatus

The subjects were 32 Lister hooded rats (Harlan) with a mean free-feeding weight of 307 g (range: 290-320), housed exactly as in Experiment 1. The experiment was run in two identical replications, and the apparatus was identical to that of Experiment 1.

### Procedure

The procedure was identical to that of *Phase 3* of Experiment 1; thus, both CS and trace interval durations were 15 s throughout. There were 16 sessions of training.

### Results

#### Difference scores

**Figure 4.**
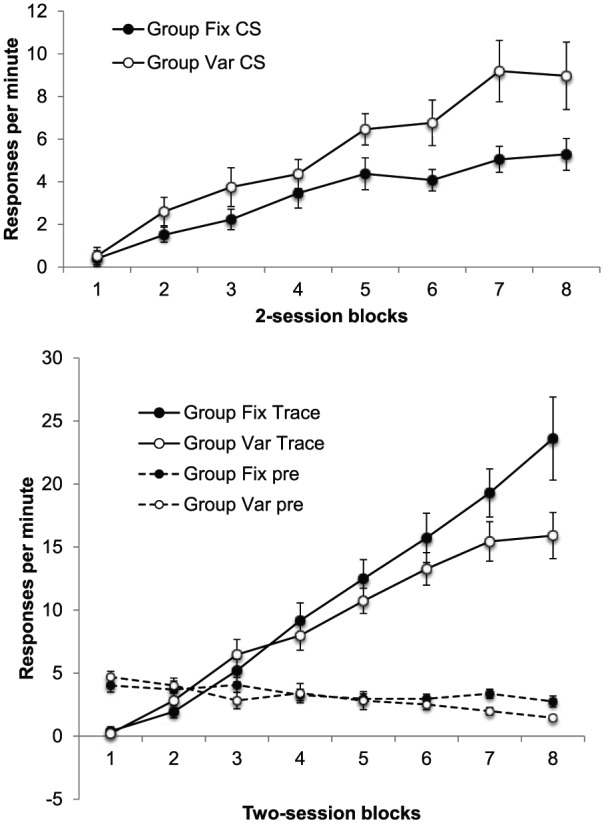
Group mean difference scores for the CS (upper panel) and the trace interval (lower panel) across training in Experiment 2; the lower panel also shows group mean rates of pre-CS responding. The data are reported in two-session blocks; error bars show standard error of the mean.

As there were 16 sessions of training, rather than 32 as in Experiment 1, the difference scores are presented in two-session, rather than four-session, blocks. These are shown in Figure 4; the top panel shows difference scores for the CS, and the lower panel shows difference scores for the trace interval and also the rates of pre-CS responding.

In Experiment 1, we found higher levels of responding in Group Fix during the trace interval, but lower responding in these animals during the CS—the opposite to the pattern we had seen in previous studies using delay conditioning tasks. This paradoxical pattern was replicated here: Figure 4 shows that there was greater responding in Group Var during the CS, but lower responding in this group during the trace interval. ANOVA with group, interval, and bin as factors yielded a significant three-way interaction, *F*(7, 210) = 7.00, *p* < .001, *MSE* = 9.59, $\eta_{p}^{2}=.19$; thus, separate ANOVAs were conducted on the CS and trace data.

ANOVA on the CS scores revealed main effects of both group and bins, *F*(1, 30) = 6.14, *p* = .02, *MSE* = 42.89, $\eta_{p}^{2}=.17$ and *F*(7, 210) = 29.00, *p* < .001, *MSE* = 6.32, $\eta_{p}^{2}=.49$, and an interaction between them, *F*(7, 210) = 2.47, *p* = .019, *MSE* = 6.32, $\eta_{p}^{2}=.08$. Responding was higher in Group Var in Bins 6, 7, and 8 (smallest, *F*(1, 240) = 5.31, *p* = .02, *MSE* = 10.89, for Block 6). ANOVA on the trace data revealed a main effect of bins, *F*(7, 210) = 73.21, *p* < 0.001, *MSE* = 6.32, $\eta_{p}^{2}=.71$, and a Group × Bin interaction, *F*(7, 210) = 3.14, *p* = .004, *MSE* = 21.47, $\eta_{p}^{2}=.10$; responding was higher in Group Fix in Bin 8, *F*(1, 240) = 13.39, *p* < .001, *MSE* = 35.42, but not on any other block (largest, *F*(1, 240) = 3.35 *p* = .07, *MSE* = 35.42, for Block 7).

Finally, ANOVA on the pre-CS rates revealed a main effect of bin, *F*(7, 210) = 6.78, *p* < .001, *MSE* = 2.44, $\eta_{p}^{2}=.18$, and a Group × Bin interaction, *F*(7, 210) = 2.10, *p* = .045, *MSE* = 2.44, $\eta_{p}^{2}=.07$; however, responding in the two groups did not differ on any block (largest, *F*(1, 240) = 3.57, *p* = .06, *MSE* = 4.29, for Block 7.

### Timing

#### Response functions

**Figure 5.**
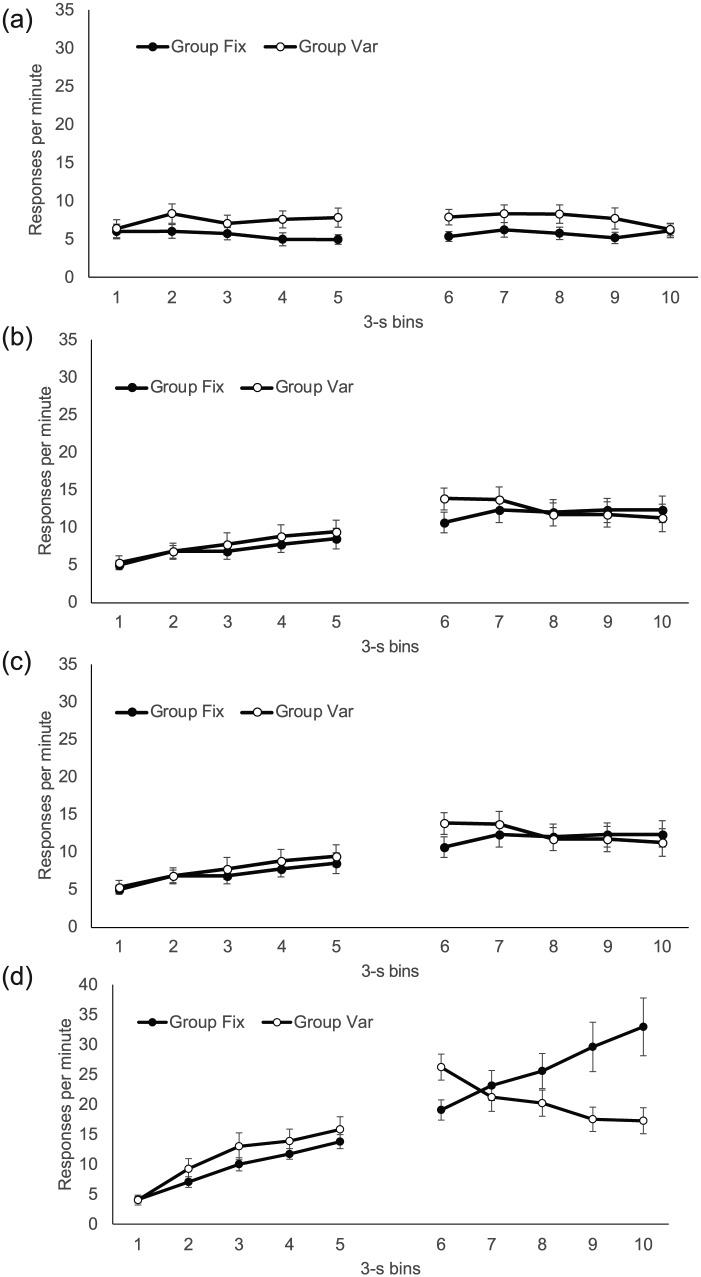
Group mean response rates in each 3-s bin of the CS and trace interval during successive four-session phases of training in Experiment 2; the first, second, third, and fourth training phases are shown from top to bottom. The CS and trace interval are five bins in all phases. Error bars show standard error of the mean.

Because we could examine the development of any timed behaviour over the course of training, the response distributions over CS and trace intervals were calculated in four phases, each comprising data from four training sessions. These data are presented in Figure 5. Response levels increased markedly over successive phases. In *Phase 1*, responding appeared higher in Group Var over *both* the CS and trace; in the CS, this pattern was maintained over subsequent blocks, but in the trace interval it gradually reversed, and Group Fix was clearly responding more than Group Var in this period in *Phases* 3 and 4.

This description was broadly confirmed by the analyses. ANOVA with *Phase* (1-4), group, interval (CS/trace), and bins (1-5) as factors revealed a significant four-way interaction, *F*(12, 360) = 9.61, *p* < .001, *MSE* = 6.27, $\eta_{p}^{2}=.24$; accordingly, ANOVAs were conducted separately on each block.

In *Phase* 1, ANOVA with group, interval (CS/trace), and bins as factors revealed a significant three-way interaction, *F*(4, 120) = 4.10, *p* = .004, *MSE* = 3.27, $\eta_{p}^{2}=.12$. ANOVA on the CS revealed a significant Group × Bin interaction, *F*(4, 120) = 3.18, *p* = .016, *MSE* = 2.67, $\eta_{p}^{2}=.1$, and Group Var responded more than Group Fix on Bin 5, *F*(1, 150) = 3.94, *p* = .049, *MSE* = 16.66; also, there was an effect of bins in Group Var, *F*(1, 150) = 3.39, *p* = .012, *MSE* = 2.67, but not in Group Fix, *F*(1, 150) = 1.69, *p* = .16, *MSE* = 2.67. ANOVA on the trace data revealed nothing significant (largest, *F*(1, 30) = 2.56, *p* = .12, *MSE*= 4.44, for the main effect of group).

For *Phase* 2, the three-way interaction was again significant, *F*(4, 120) = 5.20, *p* = .001, *MSE* = 3.71, $\eta_{p}^{2}=.15$. ANOVA on the CS data revealed only a main effect of bin, *F*(4, 120) = 17.88, *p* < .001, *MSE* = 3.81, $\eta_{p}^{2}=.37$. ANOVA on the trace interval data produced a significant Group × Bin interaction, *F*(4, 120) = 4.95, *p* = .001, *MSE* = 4.92, $\eta_{p}^{2}=.14$, but the groups did not differ on any block (largest, *F*(1, 150) = 1.86, *p* = .17, *MSE* = 43.78); however, there was an effect of bins in Group Var, *F*(1, 150) = 4.74, *p* = .001, *MSE* = 4.92, although not in Group Fix, *F*(1, 150) = 1.75, *p* = .14, *MSE* = 4.92.

The ANOVA on *Phase* 3 also revealed a significant three-way interaction, *F*(4, 120) = 19.20, *p* < .001, *MSE* = 6.49, $\eta_{p}^{2}=.39$; ANOVA on the CS data revealed only a main effect of bins, *F*(4, 120) = 56.93, *p* < .001, *MSE* = 4.27, $\eta_{p}^{2}=.66$, but the trace ANOVA yielded a significant interaction, *F*(4, 120) = 22.15, *p* < .001, *MSE* = 8.03, $\eta_{p}^{2}=.43$, and Group Fix responded more than Group Var on Block 10, *F*(1, 150) = 7.41, *p* = .007, *MSE* = 50.64; the effect of bins was significant in both groups (smallest, *F*(1, 150) = 8.03, *p* < .001, *MSE* = 8.03, for Group Fix).

Finally, in *Phase* 4, the three-way interaction was once again significant, *F*(4, 120) = 28.69, *p* < .001, *MSE* = 13.21, $\eta_{p}^{2}=.49$; ANOVA on the CS data again showed only a main effect of bin, *F*(4, 120) = 77.23, *p* < .001, *MSE* = 7.41, $\eta_{p}^{2}=.72$, whereas in the trace interval ANOVA the interaction was significant, *F*(4, 120) = 30.88, *p* < .001, *MSE* = 20.73, $\eta_{p}^{2}=.51$, and Group Fix responded more than Group Var on Blocks 9 and 10 (smallest, *F*(1, 150) = 8.91, *p* = .003, *MSE* = 131.2, for Block 4); the effect of bins was significant in both groups (smallest, *F*(1, 150) = 10.16, *p* < .001, *MSE* = 20.73, for Group Var).

In summary, this fine-grained analysis of responding over the course of the CS produced less clear an indication of higher responding in Group Var than when the data were pooled over the entire CS— although a difference was numerically evident in all four training phases, it was only significant in *Phase* 1. In contrast, there was clear evidence of a developing difference in responding during the trace interval, with Group Fix responding more than Group Var at the end of the trace interval. However, in contrast to the CS differences, this effect did not emerge until *Phases 3* and *4*.

#### Slopes

**Figure 6.**
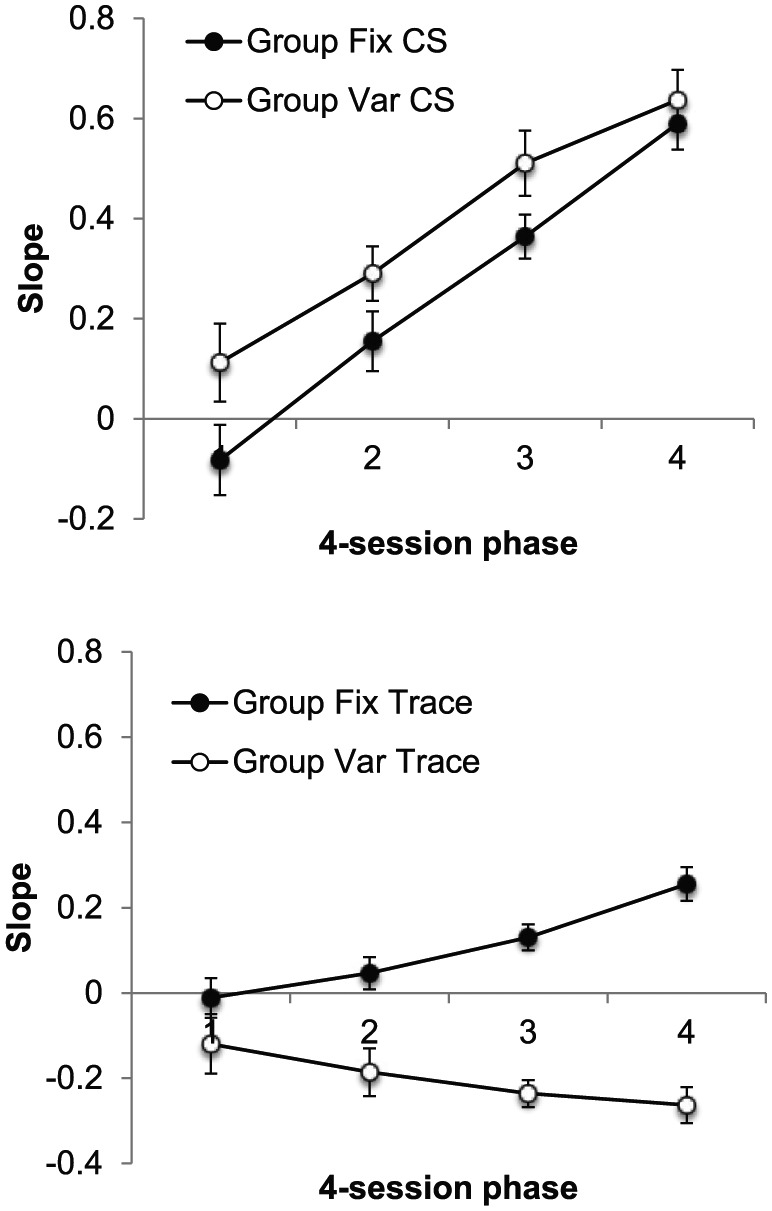
Group mean values of slope for the CS (upper panel) and trace interval (lower panel) in each four-session phase of Experiment 1. Error bars show standard error of the mean.

The group mean values of slope for the CS and trace intervals, presented separately for each phase, are presented in Figure 6. In contrast to Experiment 1, slopes were significantly higher in Group Var, suggesting better timing during the CS in these animals. As in the previous experiment, the opposite appeared to be true during the trace interval: slopes were higher in Group Fix than in Group Var and increased over bins in Group Fix but decreased over bins in Group Var.

This description was supported by the results of an ANOVA with group, interval (CS/trace), and phase as factors. This revealed main effects of group, *F*(1, 30) = 5.63, *p* = .024, *MSE* = 0.09, $\eta_{p}^{2}=.16$, interval, *F*(1, 30) = 129.96, *p* < .001, *MSE* = 0.07, $\eta_{p}^{2}=.81$, and phase, *F*(3, 90) = 33.69, *p* < .001, *MSE* = 0.039, $\eta_{p}^{2}=.53$. The critical interaction between group and interval was significant, *F*(1, 30) = 5.66, *p* = .001, *MSE* = 0.039, $\eta_{p}^{2}=.16$, and there were also significant interactions between group and phase, *F*(3, 90) = 45.40, *p* < .001, *MSE* = 0.067, $\eta_{p}^{2}=.60$, and CS and phase, *F*(3, 90) = 25.45, *p* < .001, *MSE* = 0.035, $\eta_{p}^{2}=.46$; the three-way interaction was not significant, *F*(3, 90) = 1.74, *p* = .17, *MSE* = 0.035, $\eta_{p}^{2}=.06$. Simple main effects analysis of the Group × Interval interaction revealed that the slopes were higher in Group Var during the CS, *F*(1, 60) = 7.17, *p* = .01, *MSE* = 0.068, but higher in Group Fix during the trace, *F*(1, 60) = 38.64, *p* < .001, *MSE* = 0.068.

### Discussion

These results replicated those of the previous experiment: During the CS, response levels were higher in Group Var than in Group Fix, but during the trace interval this pattern was reversed. Furthermore, these results were mirrored in the timing data. During the trace interval, timing—as indicated by higher levels of slope— was superior in Group Fix. This was to be expected as the elapsed duration of trace was a good indicator of how soon food would be delivered for these animals. But the opposite pattern— better timing in Group Var— was evident during the CS, *despite* the fact that the CS was a fixed duration in both groups.

The reciprocal pattern of conditioned responding in the CS and trace is reminiscent of overshadowing: If conditioning to the CS and trace were to compete, when conditioning to the CS is higher (as in Group Var), conditioning to the trace would be lower, and vice versa. To explore this possibility more systematically, we examined the relationship between each individual rat’s responding during these different intervals. In both Experiments 1 and 2, correlations were performed between the rates of responding during the last 3-s bin of the CS and the last 3-s bin of the trace (Figures 2 and 5). To control for overall differences in response rate between rats, partial correlations were conducted, controlling for the mean rate of responding over all 10 bins (i.e., both CS and trace). In Experiment 1, this was performed for *Phases 1, 2*, and *3*, and for Experiment 2 for all four training blocks; the Bonferroni correction was applied within each experiment. In Experiment 1, this correlation was not significant in *Phase 1, r* = –.36, was marginally significant in *Phase 2, r* = –.61 *p* = 0.05, and was significant in *Phase 3, r* = –.97 *p* < .003. A similar pattern was seen in Experiment 2, in which the correlation again failed to reach significance in Block 1, *r* = –.32, was marginally significant in Block 2, *r* = –.45, *p* = .05, and was significant in Blocks 3 and 4, *r* = –.78 and *r* = –.83 for Blocks 3 and 4, respectively, *p*s < .004. In both experiments, then, there was a significant negative relationship between responding in the CS and the trace interval that developed over the course of training. This is consistent with the proposal that the CS and trace were competing for associative strength.

However, before such a possibility can be considered, we need to confirm that the differences in responding to the CS that we have observed in these two experiments are actually due to a difference in associative strength, rather than just a performance effect. Experiment 3 examined this suggestion.

## Experiment 3

Experiment 3 was a continuation of Experiment 1 and employed a blocking design. After the end of *Phase 3*, both groups received further training in which the 15-s trace interval was removed, and a 5-s click accompanied the final 5 s of the 15-s light CS. If the CS was a better predictor of food in Group Var, then it should block acquisition of associative strength by the click more effectively, resulting in lower levels of responding to the click than in Group Fix (e.g., Mackintosh, 1975; Pearce & Hall, 1980; Rescorla & Wagner, 1972; Wagner, 1981). But if the difference in CS responding was merely a performance effect secondary to the differences seen during the trace interval, then there would be no reason to anticipate differences in responding to this added stimulus.

### Subjects

The subjects were those from Experiment 1.

### Apparatus

The apparatus was the same as in Experiment 1, except for the addition of a 75-dB 10-Hz clicker that could be delivered from the speaker.

### Procedure

#### Training

This was identical to Phase 3 of Experiment 1, except that a click of 5-s duration was presented at the end of the light CS, such that their offsets coincided and then food was delivered immediately. There were six sessions in this stage.

#### Test

This was identical to the *Training* phase except that half of the 24 trials were test trials in which the light and food were omitted: Thus, these trials comprised the 30-s pre-CS period followed immediately by a 5-s click presentation. There were four sessions in this stage. We omitted the standard control groups because the objectives of this study were not to investigate blocking during trace conditioning per se (e.g., Amundson & Miller, 2008). Rather, we wished to determine whether the differences in responding shown in Experiment 1 were due to a performance effect or a difference in associative strength. Inclusion of overshadowing controls would not achieve this objective.

#### Data treatment

Response rates during the light CS on compound trials were calculated separately for the initial 10-s period in which the light CS was presented alone and the final 5-s portion in which it was accompanied by the click. In the test phase, rates of responding during each trial type were converted into difference scores by subtracting the rate of responding during the pre-CS periods for that trial type. Pre-CS response rates were then pooled across both trial types for separate statistical analysis. The data are presented in two-session blocks. All other aspects of data treatment were identical to those of the previous experiments.

### Results

**Figure 7.**
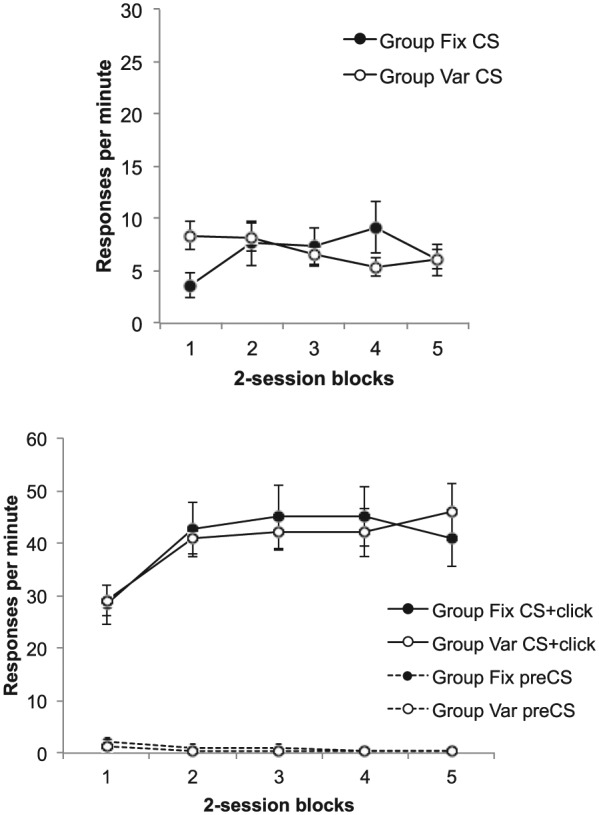
Group mean difference scores for the CS alone (upper panel) and the CS + click (lower panel) in the training trials of Phases 4 and 5 of Experiment 3; the lower panel also shows group mean rates of pre-CS responding. Data are reported in two-session blocks; error bars show standard error of the mean.

Responding during the training trials is shown in Figure 7; the upper panel shows responding during the first 10 s of the light, before the click is presented. Responding remained lower in Group Fix in the first training block, but quickly recovered to the same level as in Group Var. ANOVA revealed a significant Group × Block interaction, *F*(4, 56) = 6.54, *p* < .001, *MSE* = 5.88, $\eta_{p}^{2}=.32$, and Group Fix responded more than Group Var on Block 1, *F*(1, 70) = 4.77, *p* = .03, *MSE* = 19.27, but not on any other block (largest, *F*(1, 70) = 3.03, *p* = .09, *MSE* = 19.27, for Block 4). There also was an effect of blocks in Group Fix, *F*(4, 56) = 5.95, *p* < .001, *MSE* = 5.88, but not in Group Var, *F*(4, 56) = 2.41, *p* = .06, *MSE* = 5.88.

The lower panel shows rates of responding during the click/light compound, which was high throughout both phases, and during the pre-CS periods, which remained low; neither showed any sign of differing between the two groups. ANOVA on the click/light difference scores showed only a main effect of block, *F*(4, 56) = 10.26, *p *< .001, *MSE* = 65.57, $\eta_{p}^{2}=.42$ (other *F*s < 1). The same was true of pre-CS responding: Here, the effect of block was *F*(4, 56) = 17.18, *p* < .001, *MSE* = 0.297, $\eta_{p}^{2}=.55$, and nothing else was significant (largest, *F*(1, 14) = 2.19, *p* = .16, *MSE* = 1.57, $\eta_{p}^{2}=.14$, for the effect of group).

**Figure 8.**
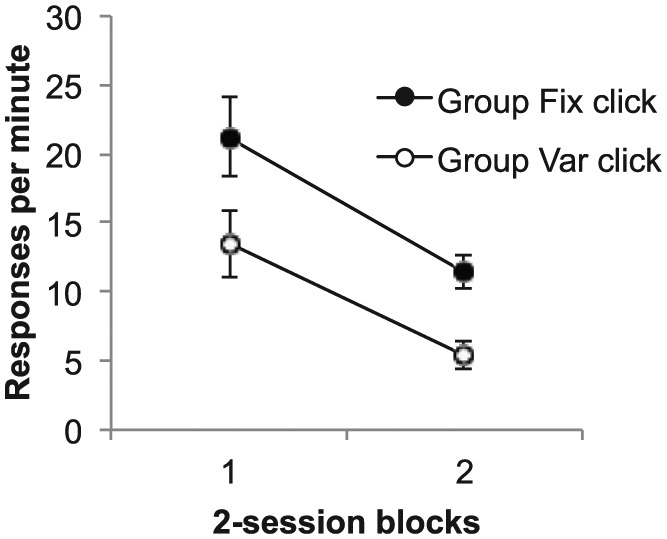
Group mean difference scores for the test trials in Phase 5 of Experiment 3. Data are reported in two-session blocks; error bars show standard error of the mean.

The key results, response rates during the test trials, are shown in Figure 8. It is clear that responding was higher to the click in Group Fix; ANOVA revealed a significant main effect of group, *F*(1, 14) = 8.49, *p* = .01, *MSE* = 44.89, $\eta_{p}^{2}=.38$; the effect of block was also significant, *F*(4, 56) = 29.44, *p* < .001, *MSE* = 21.58, $\eta_{p}^{2}=.68$, but the interaction was not, *F* < 1. Of course, in the absence of a normal overshadowing control group, these results cannot give any information about the absolute level of blocking that was obtained. Nonetheless, it is difficult to interpret the difference we did observe as other than reflecting a difference in the degree of blocking in these two conditions.

### Discussion

The results of this experiment support the suggestion that the higher responding during the CS in Group Var was due to higher associative strength, rather than some kind of performance effect. When the click was added to the end of the light CSs and this stimulus compound followed immediately by reinforcement, the click had the opportunity to acquire associative strength. However, such learning would be prevented to the extent that the light already had associative strength of its own. When the click was tested alone, responding to this stimulus was lower in Group Var than in Group Fix, suggesting greater blocking. This is consistent with the CS in Group Var having greater associative strength.

## General discussion

Experiments 1 and 2 examined learning in a trace conditioning task, in which the CS was fixed, but the trace interval was either fixed or variable. In experiments using delay conditioning, we have observed higher levels of responding during a CS that is fixed than during one that is variable (Bonardi et al., 2015; Jennings et al., 2013; Jennings & Bonardi, 2017). We have argued here that this could either be due to the temporal distribution of the CS per se or to the fact that CS onset was only informative with respect to the time of food delivery in the fixed case. The former suggestion would predict no group differences in responding during the CS in the present studies, whereas the latter would predict, as before, higher levels of responding in Group Fix. Neither result was observed; instead, significantly greater responding was seen during the CS in Group Var. Moreover, the results of Experiment 3 suggest that this difference reflected a difference in associative strength as the ability of the CS to produce blocking of the click was also superior in Group Var.

However, higher responding in Group Fix *was* clearly evident in the trace interval. If responding during a trace interval is effectively the same as responding during an actual stimulus, then this mirrors our previous findings. But then the question arises as to why the reverse effect should be seen during the CS itself. One possibility is that the CS and trace interval act as independent stimuli competing for associative strength; if the fixed trace acquires more associative strength than the variable trace, it might overshadow the fixed CS more effectively. The fact that we found significant negative correlations between responding during the end of the CS and during the end of the trace in both Experiments 1 and 2 is consistent with this suggestion.

This pattern of results leaves us no nearer to understanding the conditions under which this fixed/variable difference in acquisition of associative strength is observed: If the trace interval is effectively acting as a CS, then once again the difference could be due to its temporal distribution or the informativeness of its onset. However, the findings could have some implications for the nature of trace conditioning itself. Perhaps the simplest explanation of responding during the trace interval in these tasks is that it is a carryover of responding during the CS or responding to a trace of the CS that persists even though the CS is no longer present. But this view would expect that responding during the trace interval would reflect responding during the CS—and in our experiments this was clearly not the case: The pattern of responding quite clearly reversed between the CS and trace intervals.

There are a number of ways such reciprocity might be realised. One obvious suggestion is to appeal to the context: Contextual cues are present during the trace interval, and these can also acquire associative strength. If there were competition between the CS and the contextual cues for associative strength, this could produce the observed pattern of results. Specifically, one could suppose that the contextual cues, being most proximal to reinforcement, acquire associative strength first. When the trace interval is of a fixed duration, these contextual cues acquire more associative strength than when the trace is variable, exactly as was observed in our previous delay conditioning studies. The more associative strength the contextual cues acquire, the more effectively the trace CS would be overshadowed—hence the reciprocity in the pattern of responding during trace CS and trace inteval.

But whatever the merits of this account, the high level of responding during the trace interval requires some explanation; because the context is present throughout the session, there is more than ample opportunity for these cues to extinguish. Moreover, even if the contextual cues were able to acquire strength in the manner described, without extra assumptions there should be a similar pattern of responding during the pre-CS periods—yet responding in the pre-CS periods was negligible and largely similar in the two groups. One way out of this paradox relies on summation: Subthreshold differences in contextual associative strength might only become evident when they summate with the CS’ associative strength. Another is the idea that trace conditioning engages a form of occasion setting, in which the CS is an occasion setter signalling reinforcement of the context (e.g., Brown, Hemmes, de Vaca, & Pagano, 1993). This could explain why the context commanded such higher levels of responding only when preceded by the CS. As occasion setters’ properties are exercised independently of their own associative strength (see Bonardi, Robinson, & Jennings, 2017 for a recent review), this could allow a different pattern of responding during the CS and trace. Conversely, the effect could be explained by theories of conditioning that suppose the trace of a CS that persists after its offset are qualitatively different from the CS itself and can support discriminable patterns of behaviour (e.g., Brandon, Vogel, & Wagner, 2003; Lin & Honey, 2011).

Finally, these results might also be accommodated by models that stress the importance of memory in determining response rates (e.g., Gibbon, Church, & Meck, 1984). For example, scalar expectancy theory argues that the decision whether or not to respond within a trial is based on the amount of elapsed time on the current trial, in relation to the remembered time of US from previous trials (Gibbon, 1977). In this study, two groups received different temporal arrangements of the trace interval. Where both the CS and trace were fixed in duration, the onset of the CS signals the time to reinforcement. Even without appealing to the (highly) salient time marker of CS offset/trace onset, responding is expected to increase as US delivery becomes imminent (e.g., Jennings et al., 2013). However, where a fixed-duration CS is followed by an exponentially distributed trace duration, the remembered time to reinforcement should differ from a fixed duration. This is because exponential distributions draw a majority of trial durations that are shorter than the mean (Kirkpatrick, 2002). If subjects remember specific rather than averaged trial durations (Gibbon, Church, Fairhurst, & Kacelnik, 1988), the preponderance of remembered short durations may result in high responding at trace onset with a rapid decline in responding as the time to US delivery increases (see Harris, Gharaei, & Pincham, 2011). Thus, the discrepancy in responding between the two groups in both the CS and trace might be accounted for by appealing to differences in the remembered times to reinforcement.

In summary, the present experiments represent an empirical extension of our previous findings. Specifically, we show that fixed duration intervals that signal the delivery of reinforcement elicit higher levels of responding than variable duration intervals; although in our previous experiments these intervals were CS presentations, in the current experiments they corresponded to the trace interval of a trace conditioning task. Moreover, a reciprocal pattern was seen during the CS, suggesting that the CS and trace were in competition for associative strength. These findings extend our understanding of the effects of temporal factors on conditioning and are compatible with accounts of trace conditioning that view the trace as qualitatively distinct from the CS.
